# Social Listening to Enhance Access to Appropriate Pandemic Information Among Culturally Diverse Populations: Case Study From Finland

**DOI:** 10.2196/38343

**Published:** 2022-07-08

**Authors:** Anna-Leena Lohiniva, Katja Sibenberg, Sara Austero, Natalia Skogberg

**Affiliations:** 1 Finnish Institute for Health and Welfare Helsinki Finland

**Keywords:** infodemic, social listening, pandemic preparedness, cultural diversity, vulnerable populations

## Abstract

**Background:**

Social listening, the process of monitoring and analyzing conversations to inform communication activities, is an essential component of infodemic management. It helps inform context-specific communication strategies that are culturally acceptable and appropriate for various subpopulations. Social listening is based on the notion that target audiences themselves can best define their own information needs and messages.

**Objective:**

This study aimed to describe the development of systematic social listening training for crisis communication and community outreach during the COVID-19 pandemic through a series of web-based workshops and to report the experiences of the workshop participants implementing the projects.

**Methods:**

A multidisciplinary team of experts developed a series of web-based training sessions for individuals responsible for community outreach or communication among linguistically diverse populations. The participants had no previous training in systematic data collection or monitoring. This training aimed to provide participants with sufficient knowledge and skills to develop a social listening system based on their specific needs and available resources. The workshop design took into consideration the pandemic context and focused on qualitative data collection. Information on the experiences of the participants in the training was gathered based on participant feedback and their assignments and through in-depth interviews with each team.

**Results:**

A series of 6 web-based workshops was conducted between May and September 2021. The workshops followed a systematic approach to social listening and included listening to web-based and offline sources; rapid qualitative analysis and synthesis; and developing communication recommendations, messages, and products. Follow-up meetings were organized between the workshops during which participants could share their achievements and challenges. Approximately 67% (4/6) of the participating teams established social listening systems by the end of the training. The teams tailored the knowledge provided during the training to their specific needs. As a result, the social systems developed by the teams had slightly different structures, target audiences, and aims. All resulting social listening systems followed the taught key principles of systematic social listening to collect and analyze data and used these new insights for further development of communication strategies.

**Conclusions:**

This paper describes an infodemic management system and workflow based on qualitative inquiry and adapted to local priorities and resources. The implementation of these projects resulted in content development for targeted risk communication, addressing linguistically diverse populations. These systems can be adapted for future epidemics and pandemics.

## Introduction

### Background

An infodemic is defined as an overabundance of information, some accurate and some not, which occurs during an epidemic [1]. During the COVID-19 pandemic, the infodemic has been rapidly expanding and evolving, particularly via social media channels. It has been estimated that rumors are 3 times more likely to be spread via social media than accurate information [2]. An infodemic poses a challenge for public health authorities who must continuously produce trustworthy and relevant information to inform the public about risks, influence behavioral change, and encourage compliance with emergency measures. Successful infodemic management saves lives and ultimately plays a major role in pandemic mitigation efforts. In contrast, failures in infodemic management during a pandemic can lead to misinterpreted messages, failed warnings, false rumors, and inconsistent information, which can negatively influence adherence to preventive behaviors of the public, which can be life-threatening and can negatively affect the trajectory of the pandemic [1,3]. Numerous infodemic examples have been documented during the pandemic, some of which have permeated geographic, cultural, and socioeconomic boundaries and required health care resources that, in most parts of the world, were already limited because of the pandemic. For example, a rumor that consuming highly concentrated alcohol could disinfect the body and kill the coronavirus [4] led to hospitalizations and fatalities after people ingested methanol in several countries, including Iran, Turkey, India, South Korea, and Qatar [5-8]. In addition, conspiracy theories circulated widely across the globe, often intentionally spreading disinformation [9].

Similarly, Finland witnessed a widespread infodemic during the COVID-19 pandemic. For example, anecdotal data point out that at the beginning of the COVID-19 vaccination campaigns, there were rumors that the vaccines were offered to harm people of particular ethnic backgrounds. Other widespread rumors claimed that the pandemic was created by pharmaceutical companies to make money by selling vaccines or that certain countries were responsible for the pandemic [10].

The World Health Organization (WHO) infodemic management framework advances equity as it highlights the importance of social listening and the need to identify context-specific information to tailor culturally appropriate infodemic responses [11]. Context specificity in infodemic management is of utmost importance as the pandemic has disproportionately affected ethnic minorities and a broad range of other populations that were already at a social disadvantage before the epidemic [12]. Members of minority groups may also be more resistant to following the guidance of authorities as they are often economically and socially more vulnerable than others [13]. Moreover, effective communication with various population subgroups tends to require tailored approaches [14].

Finland has become increasingly culturally and linguistically diverse in the past few decades. At the end of 2020, people speaking languages other than the official languages of Finnish, Swedish, or Sami constituted approximately 8% of the total Finnish population [12]. The increasingly linguistically and culturally diverse environment adds to the complexity of risk communication disseminated by health authorities. As in many other high-income countries, individuals of migrant origin in Finland were reported to have a higher incidence of COVID-19 infections and lower vaccine uptake than the general population [12,15]. This raised the need to better inform and engage people of migrant origins in risk communication planning and dissemination. Occasionally, individuals of migrant origin have also received negative attention and have been stigmatized as careless and unwilling to follow the guidance of health authorities, which has also raised awareness about the need to gain a better understanding of how various subgroups think. During the pandemic, health authorities have frequently highlighted the need for equity in health information and access to acceptable and appropriate information for everyone.

Social listening, a continuous process of collecting web-based and offline data using standard tools, has increased during the pandemic. Social listening projects have taken many forms worldwide. For example, in Vietnam, a social listening project was set up to explore public attention toward the pandemic, whereas another recent study concentrated on English-language tweets to identify the main pandemic topics globally [16,17]. Some projects use big data and dashboards to present the findings, such as the Red Cross COVID-19 dashboards piloted in some countries and the WHO Early Artificial Intelligence–Supported Response with Social Listening that monitors COVID-19–related web-based discussions in 30 countries [18-21]. Other projects have focused on smaller data sets based on manual internet browser searches and qualitative methods [3,22]. In addition, some projects have collected field-based data on rumors such as a real-time rumor-tracking pilot in Côte d’Ivoire, which leverages existing structures, including hotlines and community health workers, to submit rumors to a central database for rapid coding and visualization of the findings on dashboards [23].

In February 2020, the Finnish Institute for Health and Welfare (*Terveyden ja hyvinvoinnin laitos*; THL) initiated a social listening project to monitor pandemic perceptions of the public to provide recommendations for public authorities and risk communicators. The process comprised qualitative data collection and analysis in real time based on social media posts and information inquiries emailed by the public to THL. The results were shared and discussed with a group of public health and risk communication experts to determine appropriate infodemic responses every 2 to 4 weeks [3]. In May 2021, the project expanded to include training for regional health authorities and nongovernmental organizations that communicate COVID-19–related information to culturally and linguistically diverse populations. The extended social listening training project was initiated in response to public health experts’ frequent concerns that pandemic-related information may not be reaching all population groups equally in Finland. Although community outreach workers have been listening to their target audiences even before joining the extended social listening project, there has not been a systematic and structured way of collecting, analyzing, and using the data. THL’s multilingual and multichannel communication task force implemented the expanded social listening project with the goal of supporting organizations in designing targeted communication for people from various ethnic backgrounds during a crisis.

### Objectives

This study describes the development of systematic social listening training during the COVID-19 pandemic through a series of web-based workshops. It also reports the experiences of the workshop participants in implementing the projects.

## Methods

### Training Workshops

The overall concept behind the social listening workshops was that generic risk communication messages are not effective in reaching various subpopulations or changing the behaviors of target groups. Accordingly, having a systematic process that helps health authorities better understand the needs and motivations of various subpopulations will lead to more effective communication that is likely to lead to more sustainable behavior changes. Context-specific messaging also ensures less misunderstanding between health authorities and the public, which may help build increased trust between the two [24]. The training workshops were based on the conceptual framework of the WHO in infodemic management, which includes social listening, translating knowledge to practice, and quantifying impact [4] to expel misinformation and support targeted communication during crises.

The workshops were designed by a multidisciplinary team of experts from the THL. An anthropologist with a background in behavioral sciences and experience in social listening was mainly responsible for the content of the training workshops. An expert in pedagogy and risk communication was responsible for the contents of risk communication and for the overall structure of the workshops, including the timing and methods applied in group exercises. In addition, an expert in migration and cultural diversity was the main coordinator who also critically reviewed the social listening projects, ensuring that they were culturally appropriate.

The workshop design took into account the pandemic context. For example, training had to be short and intensive for participants to have time to participate. The aim of the training was to provide participants with sufficient knowledge and skills to develop their own social listening project based on their priorities, systems, and available resources. The workshops were based on a careful mix of tools that promoted the participation and internalization of knowledge and its application in a real-life project. The workshop structure was based on the principles of active learning [25]; for example, the rather rapid pace of alternating between activities aimed at maintaining active learning among the participants and motivating them to continue the training. The workshop methodology also used team-based learning pedagogy by introducing a systematic approach to building social listening projects in teams [26]. The workshop used Microsoft Teams and additional digital platforms, such as Howspace, for group exercises and for compilation of all workshop materials and suggested materials for further learning that participants could access after the training workshops as well.

The design also included homework that was meant to allow participants to practice what they had learned during the workshop and thus advance in developing their own social listening project design. The expected outcome of the workshops was a draft project plan by each team, including project flow, goals and objectives, data collection, and an analysis plan. The social listening methodology was based on qualitative data collection and analysis, with the notion that in-depth qualitative data provide a rich base for risk communication content development [27]. However, qualitative methods can be time consuming and complex [28], which requires adapting rapid qualitative data collection, recording, and analysis methods. Accordingly, the workshop encouraged the participants to adapt and test the taught methods of social listening to identify the best possible type of data collection and analysis for their specific needs. During the workshops, a substantial amount of time was allocated to teaching strategies on how to simplify the qualitative data collection and analysis processes.

The social listening training was designed for individuals responsible for community outreach work and for communication disseminating information to culturally diverse populations. The THL’s multilingual and multichannel communications task force invited their collaborators from various cities and the Finnish Red Cross (FRC), which coordinated multilingual and multichannel projects among 20 local nongovernmental organizations. The invitation included a request to form a team that included those who could collect and analyze data and those who could develop communication messages and products. Each team was requested to have outreach workers and at least one communication expert.

### Experiences in Implementing Social Listening Projects

Data on experiences in social listening project implementation were collected from the final presentations that each team shared at the end of the training, followed by 30-minute, one-on-one telephone interviews with each participant conducted by the corresponding author (ALL) in November 2021. Teams that did not continue implementing social listening followed by the workshops were requested to explain the reasons for this. The author also analyzed the interviews thematically using NVivo (QSR International) and shared the findings with the teams for verification [29].

### Ethics Approval

Social listening was implemented using publicly available data that did not contain personal or sensitive data. Each team conducting social listening complied with ethical considerations based on the guidance from their own institutions (City of Helsinki, City of Espoo, City of Vantaa, and FRC). All teams ensured confidentiality through the anonymization of their data. No personal identifiers were collected. Confidentiality was maintained throughout the entire project cycle from data collection to reporting.

## Results

### Workshops

**Figure 1:**
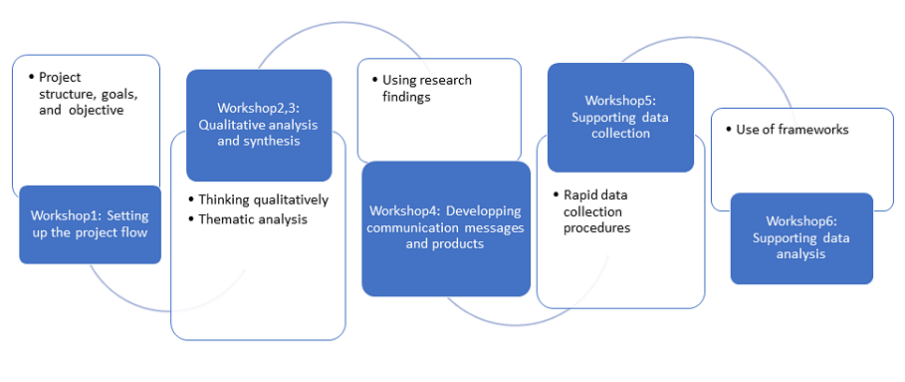
Structure of the training workshops.

A series of 6 web-based workshops was delivered between May and September 2021, by a multidisciplinary team of 3 experts from THL responsible for the methodological design of the workshops. The time between the first 4 workshops ranged from 1 to 2 weeks. Workshops 5 and 6 were conducted after the summer holidays, resulting in a nearly 2-month break from the earlier workshops. Each workshop lasted for a maximum of 2 hours and included short lectures, discussions, and exercises. Between the workshops, participants were assigned homework that focused on the implementation of the techniques learned during the workshops. The workshops were developed based on the following structure: (1) setting up a social listening project with roles and responsibilities and defining goals and objectives; (2) learning about the use of qualitative methodology and how to think qualitatively; (3) qualitative analysis and synthesis; (4) developing communication recommendations, messages, and products; (5) focusing on how to facilitate qualitative data collection procedures; and (6) learning to facilitate qualitative data collection procedures. A total of 2 consultations were organized by the trainers in between the workshops, during which participants shared their achievements and challenges. All team members were invited to the consultations (Figure 1 provides the structure of the training workshops).

All 6 workshops were conducted on the web with a carefully planned set of learning objectives, themes, and structures to ensure that they kept participants interested and occupied during the training. Accordingly, the workshop structure was based on short activities that started with a theory session followed by interactive group work. During the group work, teams were required to apply the theory through reflections and discussions to ensure that the theory and its application were truly internalized. Group work was always conducted within the participants’ own social listening teams, with the exception of qualitative analysis in which each individual practiced data coding themselves. The participants also completed 3 homework assignments in between the workshops. Each team presented their homework to the others during the following workshop. A detailed plan of the workshops is presented in Table S1 in Multimedia Appendix 1.

The workshop participants also received a list of resources that they could use to deepen their understanding of the methods, techniques, and conceptual frameworks that were introduced during the workshop, as shown in Multimedia Appendix 2.

### Social Listening Projects

#### Overview

A total of 6 social listening teams joined the workshops from different geographic locations in Finland. A total of 4 groups implemented the project. Of the 2 groups that dropped out, one did not have sufficient human resources to conduct the project, whereas the other group did not know how to reach their target audience. The remaining teams comprised outreach workers and communication professionals. The participants had little or no prior experience in applying the research methods in their work. The following section describes how the teams formulated their social listening processes following the training. A summary of these projects is provided in Table 1. Details of the social listening team composition and the resources invested are provided in Table 2.

**Table 1:** Summary of social listening projects.

Project	Objectives	Data sources	Methodology
City of Helsinki	To identify key concerns among the public to prepare and disseminate appropriate information To detect and correct misinformation	A number of social media channels and face-to-face encounters with clients	The weekly manual process included reviewing posts and extracting relevant posts to a spreadsheet followed by team discussions about the type of communication actions needed
City of Vantaa	To track the main discussions and themes related to the pandemic and COVID-19 vaccines	Face-to-face encounters with clients	Data collection through discussions and field-based observations by outreach workers Project team meeting once a month to share, analyze, and brainstorm how to use the observations, followed by the development of messages and materials that are distributed through known channels to target audiences
Espoo	To create vaccine demand	Face-to-face with client encounters and social media	Informal discussions conducted during routine meetings are used to develop communication responses
Finnish Red Cross	To develop targeted communication materials	Face-to-face discussions with partner organizations working with different language groups	Partner organizations shared their experiences of encounters with different language groups with Finnish Red Cross project management who develop communication materials based on those encounters

**Table 2:** Average time and human resources spent on social listening.

Participants	City of Espoo	City of Helsinki	City of Vantaa	Finnish Red Cross
The number of people who participated in the training	6	6	9	3
Number of people involved in data collection	6	5	12-20	2
Number of people involved in data analysis	4	6	12	2
Number of communication experts	2	0	4	1
Time per week spent on the social listening project per person, mean (SD)	+1 or –1	N/A^a^	+1 or −1	4

^a^N/A: not available.

#### City of Helsinki

The social listening projects of the city of Helsinki aimed to listen to the information needs of people from linguistically diverse populations. The team comprised social media experts, whose data sources included one social media site run by the city of Helsinki and several open sites where linguistically diverse populations communicate. In addition, data were collected during face-to-face encounters with their clients, such as at the information desk of the Helsinki main library. Each data source had a focal point who recorded data independently on their work laptop and summarized the data into a joint Microsoft Excel sheet located on a secured server that could be accessed only by the team members. No identifiers were collected. The content of the sheet was discussed on a weekly basis to guide the planning of information provision for linguistically diverse populations. During the piloting period, social listening identified critical information voids that were addressed in open webinars and pop-up consultations that the city was organizing to boost the COVID-19 vaccine uptake. This project has opened new social media channels that can be included in future social listening activities.

#### City of Vantaa

The social listening project of the city of Vantaa focused on listening to the COVID-19 vaccine and pandemic-related discussions among linguistically diverse populations. The social listening project team comprised a communication expert specializing in multilingual communication and approximately 20 outreach workers with diverse cultural backgrounds across the city who work with linguistically diverse populations, such as groups of migrant origin, or with projects targeting audiences of all major language groups under one focused theme such as employment creation. Data collection was based on face-to-face encounters with target audiences and manual notetaking during these encounters. Each outreach worker was responsible for keeping the notes in a secure location at the workplace. The notes included no names or any other identifiable information. Social listening was introduced as a continuous process, not based on any specific schedule or weekly time limit but on ad hoc opportunities to chat with the target audience. The social listening group met monthly to share, analyze, and reflect on field observations on an agreed-upon topic through an open discussion that culminated in jointly agreed-upon communication messages and actions. During the piloting period, social listening pinpointed a number of factors that influenced COVID-19 uptake among Russian-speaking and Somali-speaking clients, which were used to develop targeted messages for discussion events organized by the city. At the beginning of the process, the team had a joint platform to record data that were later omitted from the process as it was too time consuming. The project staff highlighted that the project was beneficial for the team members as it opened up opportunities for outreach workers to influence communication and for the communication experts to develop context-specific messaging. Following the pilot, the group plans to continue the project by improving the working modalities and developing checklists that can better focus on observations and analysis in the future.

#### City of Espoo

The city of Espoo introduced the social listening model to a number of different working groups and projects that communicated pandemic-related information to linguistically diverse populations; however, it did not formalize the system. Instead, social listening is considered a tool that can be used periodically when needed. Outreach team members verbally discussed the outcomes of encounters with their clients regarding the pandemic during a routine biweekly meeting to improve the messages that they communicated. Social listening was based on recall, and no notes were taken. The team members highlighted that the COVID-19 vaccination program has benefited from the social listening system by using the findings to create content for their COVID-19 and vaccination webinars. The process is still in the testing phase; however, future plans include formalizing social listening and creating a more formal structure to help monitor the process.

#### The FRC Organization

Social listening was part of a multilingual communication project of the FRC. The project aimed to develop pandemic-related multilingual materials based on data gathered by the FRC district offices and >10 partner organizations across the country. Partner organizations included 2 large umbrella organizations that covered a number of smaller organizations, all of which were in direct contact with people from culturally and linguistically diverse backgrounds. The focal points of the organizations were brought together with the FRC project coordinator to share their observations. The system was built upon the partners’ requests for no formal structures, written data collection, or documentation, and it used existing meetings of the partners, which ensured minimum use of resources. Data collectors were not requested to take notes or use a specific amount of time for observations, and they shared insights based on what they memorized at the time of the meeting. All discussions were confidential. The observations were discussed, and materials were developed and shared with the focal points for their feedback before finalization. The focal points could also be consulted on a one-on-one basis to seek their opinions about certain messages and materials. The system has regularly fed into communication content and FRC, which develops materials, and their partners have realized the benefit of discussing topics of interest before they are implemented as communication messages and materials. The new working modalities are expected to expand to include other topics and collaborations.

## Discussion

### Principal Findings

This study provides valuable insights into a series of rapid social listening workshops designed to provide training participants with the knowledge and skills to develop their own social listening systems based on qualitative data collection and analysis. To our knowledge, this is the first paper describing the structure and contents of social listening training that focused on qualitative data analysis and synthesis and targeted individuals without previous research experience, who were responsible for conducting community outreach work. This paper further demonstrates how the training workshop participants adapted the knowledge and skills gained in the workshops in different contexts for implementing social listening programs among culturally and linguistically diverse populations during a crisis. The projects had different aims, target audiences, data sources, and working modalities; however, they were all able to produce meaningful insights that were further used to develop acceptable and appropriate communication messages for people belonging to different cultural and linguistic groups.

Approximately 67% (4/6) of teams that continued with designing their own social listening projects completed all the offered training workshops, designed their own social listening plans, and then successfully implemented these plans. A web-based methodology with short but focused sessions made participation logistically easier. Distant learning may have been even a prerequisite for participation for some of the participants in the midst of their hectic pandemic work schedule. However, at the same time, the training participants did not have the opportunity to learn about one another. Thus, informal learning between the teams that often occurs during coffee break discussions in face-to-face training was lacking. It is likely that in future crisis situations, similar training should also be conducted on the web. However, more mixing of the teams during group work exercises and reflection sessions could be embedded in the workshops to facilitate peer reflection and learning across different teams. The trainers did not mandate the workshop participants to keep their cameras on during the training. However, in the future, training workshops could be mandatory to foster communality.

The social listening methodology introduced in this project was based on qualitative inquiry, which is often perceived as difficult to implement [28]. Accordingly, during the workshops, a substantial amount of time was spent learning about the importance of qualitative data and various modalities that can be used to simplify the processes. The findings showed that projects had adopted rapid but systematic data collection and analysis processes, including the use of recording data in a joint Microsoft Excel sheet, handwritten notes, or memorizing data and verifying data weekly or monthly in a joint meeting. Interestingly, digital platforms such as Microsoft Teams, Google Docs, or other technological tools such as voice messaging were not widely used when implementing the projects. In contrast, some projects found them more time consuming than traditional paper and pen notetaking. Two projects used a joint platform where individuals organized their data for discussion. The use of joint platforms to display data has been commonly used in other social listening projects, such as in Kenya, which had a messaging matrix available for all who communicate. In Sierra Leone, a cloud-based data collection resulted in a real-time message dashboard [2,30].

However, the projects did not use any particular behavioral frameworks or checklists that were introduced during the training, which were meant to facilitate data collection and analysis processes. It would be important to investigate the reasons for this as they have been found to be helpful in social listening projects elsewhere. For example, this was the case in Côte d’Ivoire, where phone hotline–based data managers coded rumors nearly in real time according to behavioral theory frameworks [23]. In addition, the projects did not develop procedures that would show how the data were interpreted or synthesized. Knowing that rapid and rigorous data analysis is a particularly daunting task, more tools could be introduced in future training. Such tools may be, for example, a rigorous and accelerated data reduction technique that converts raw textual data into a more manageable and user-friendly format, which involves systematic analysis during each step of the process [31]. As all training materials were provided to the trainees, the use of frameworks and improved recording of data analysis are issues that the teams can also develop later on once they want to start improving their methodology and systems.

Knowledge co-creation was a key feature of each project, highlighting the understanding of the essence of qualitative approaches that appreciate reflection [32]. All projects invested time to discuss the findings and to co-create messages and products, which is likely to ensure that the findings and resulting recommendations were seen by the target audience as salient, legitimate, and credible [33]. Knowledge co-creation also promoted shared learning, which is likely to result in more impact-driven risk communication [34,35]. Knowledge co-creation further emphasized the importance of a multiprofessional composition [36] of the teams, including field-based data collectors who have direct contact and access to target populations and communication experts with the ability to produce quality messages and materials. One of the teams mentioned that the collaboration between field teams and communication experts was an entirely new experience that was beneficial for both parties.

Examples of training participants’ use of social listening data in communication with people from linguistically diverse backgrounds indicate that they internalized the very essence of cultural relativism, namely, valued the ideas of the target audience instead of judging them against expert opinions [37,38]. They used the thinking of their target audience to create communication that facilitated 2-way dialog. The realization of a lay perspective is also likely to decrease potential misunderstandings that are common when scientific or expert information is communicated to the general public [39]. Generic messages are rarely effective in changing attitudes and behaviors, unlike focused messages, which are based on an understanding of the target audience’s needs [40,41].

All projects avoided highly structured systems in favor of informal and flexible approaches to make the data collection and recording process less time consuming and complex. Flexible structures are more adaptable to changing topics and target audiences, which is highly beneficial for social listening projects that aim to provide real-time information about relevant topics. Formal structures are likely to be seen as commitments that the organizations are not willing or able to make without a dedicated budget that none of them had for social listening purposes. The more flexible social listening projects were merged within their structures and ongoing activities, the more cost-effective the projects were. A formal structure would allow the institutionalization of social listening as a part of routine risk communication during future crises [42].

All teams demonstrated having developed targeted communication materials based on social listening after attending the workshops. These included content for webinars, pop-up consultations, face-to-face meetings, and printed materials. Pilots from other parts of the world with similar social listening projects, which have triangulated insights from digital and nondigital sources, have also developed meaningful communication; however, impact evaluations have not been conducted [43,44]. As 2 out of 6 teams dropped out without piloting social listening, it would be important in the future to better define the selection criteria to participate in the workshops and follow up on the selection of the workshop participants. Future plans could involve the development and testing of a joint platform across organizations that can share real-time data for communication purposes. Thus far, there has been no monitoring system to track changes in the attitudes or behaviors of target audiences. In the future, it would be important to integrate rigorous monitoring and evaluation components into projects to understand how targeted messaging influences the audience. It is also important to continue testing and learning from different project modalities. More efforts should be made to increase the use of multiple data sources to establish an integrated analysis that can further strengthen the quality of the data analysis and the recommendations [1].

### Conclusions

A series of training workshops was designed to implement social listening based on qualitative data collection and analysis for individuals responsible for community outreach and for communication specialists who had little or no prior experience in research methods. Over the course of the training, the participants adapted the frameworks and techniques introduced during the training to design their own adapted social listening systems. These social listening systems were based on their specific priorities and resources. The implementation of these systems resulted in content development for targeted communication messages addressing linguistically diverse populations. They can be adapted for use in future epidemics and crises. Future studies should aim for more long-term follow-up of the implementation and impact assessment of the projects.

## Appendix

Multimedia Appendix 1 The curriculum of a series of workshops preparing participants to develop social listening projects.

Multimedia Appendix 2 Resources for trainees.

## Abbreviations

FRC: Finnish Red Cross
THL: Terveyden ja hyvinvoinnin laitos (Finnish Institute for Health and Welfare)
WHO: World Health Organization
