# Supplementary material for: Social Listening to Enhance Access to Appropriate Pandemic Information Among Culturally Diverse Populations: Case Study From Finland
Source: JMIR Infodemiology. 2022 Jul 8;2(2):e38343. doi: 10.2196/38343 (PMC10014086; doi:10.2196/38343)
Supplement: Multimedia Appendix 1 [file infodemiology_v2i2e38343_app1.docx]

**Multimedia Appendix 1.** The curriculum of a series of workshops preparing participants to develop social listening projects.

| Name of the workshop | | Learning objectives | Key themes | Training outline |
| --- | --- | --- | --- | --- |
| **Workshop 1** | | | | |
|  | Project flow-social listening | - To define social listening - To clarify the benefits of social listening - To develop a social listening system | - Social listening is a structured and systematic process - The context matters (to understand and to be understood) - Social listening reveals factors that drive the behaviors of individuals; risk perception and motivations - Knowledge does not change behavior - Effective communication is developed jointly with the target audiences based on their priorities, needs, and concepts | - Welcome and agenda: a short round of introductions of each team and expectations (15 minutes) - PowerPoint presentation: What do we mean by social listening (20 minutes) - Group work: discuss how your team can benefit from social listening (15 minutes) - PowerPoint presentation: examples of social listening projects (20 minutes) - Group work: decide on the aim and the target audience of your project, and develop a project flow (30 minutes) - Reflections about the group work, introduction to homework, and closing (20 minutes) - Total time: 2 hours |
|  | Homework | - To develop/finalize my own project design to be presented in the following workshop | - Aims and objectives - Target audience - Data sources - Roles and responsibilities - Working modalities - Schedule | —^a^ |
|  | Expected outcome | - Teams develop a short overview of their project, including all key elements that allow them to start planning for the implementation of the project | - Teams develop a short overview of their project, including all key elements that allow them to start planning for the implementation of the project | - Teams develop a short overview of their project, including all key elements that allow them to start planning for the implementation of the project |
| **Workshop 2** | | | | |
|  | Basics of qualitative research and identification of keywords | - To recognize the basic principles of qualitative inquiry that can be applied in social listening - To develop keyword searches for social listening projects using various theoretical frameworks | - Learning to think qualitatively is required when doing qualitative analysis - There are benefits and limitations of qualitative inquiry in social listening, which are important to realize - The use of keywords and other structured approaches make qualitative analysis less time consuming | - Welcome and agenda: each group makes a short introduction to project presentations (homework); feedback is provided by the facilitators (45 minutes) - Interactive PowerPoint presentation: the fundamentals of qualitative research; during the presentation, participants can place questions in the chat box (20 minutes) - PowerPoint presentation: how to use keywords in social listening (15 minutes) - Group exercise: how to link project aim, research questions, and keywords in the social listening project (30 minutes) - Reflections, introduction to homework, and closing (10 minutes) - Total time: 2 hours |
|  | Homework | - To include key questions and keywords for the data analysis process, which will help to focus the project | - Key questions - Keywords | — |
| Merge | Expected outcome | - Teams develop their data collection plan reflecting their projects objectives | - Teams develop their data collection plan reflecting their projects objectives | - Teams develop their data collection plan reflecting their projects objectives |
| **Workshop 3** | | | | |
|  | Qualitative data analysis | - To apply qualitative coding and synthesis in social listening | - To conduct coding and categorization of qualitative data using social listening data using rapid analysis methods - Analysis can be simplified - Synthesis of data from different data sources requires logical thinking | - Welcome and agenda: homework (each group makes a short presentation); facilitators provide feedback (40 minutes) - PowerPoint presentation: qualitative analysis (20 minutes) - Individual exercise: coding and categorizing text (20 minutes) - Large group discussion about the exercise and closing (40 minutes) - Total time: 2 hours |
|  | Homework | - To plan for a coding system of social listening data | - Specifying what will be coded, how it will be coded, who will be coding, and how data interpretation will be conducted | — |
|  | Expected outcome | - Teams develop their own data analysis plan | - Teams develop their own data analysis plan | - Teams develop their own data analysis plan |
| **Workshop 4** | | | | |
|  | Applying social listening data in risk communication | - To use findings of the social listening process in risk communication | - Risk communication principles | - Welcome and agenda: homework (each group makes a short presentation); facilitators provide feedback (40 minutes); reflections and closing - PowerPoint presentation: risk communication and use of social listening data (20 minutes) - Group work: developing recommendations for risk communication based on social media data (20 minutes) - Reflections on the group work and open discussion about how the groups plan to take off with the social listening plans (20 minutes) - Total time: 1 hour 40 minutes |
|  | Expected outcome | - Teams design communication messages or products based on their social listening data | - Teams design communication messages or products based on their social listening data | - Teams design communication messages or products based on their social listening data |
|  | Consultation | - To follow up on progress and provide support | - Social listening is easy - Context can pose challenges and opportunities - Projects can be adjusted based on the context | - Welcome and agenda - Short presentations of all projects; one thing that has worked very well; facilitators provide feedback (45 minutes) - Identifying needs for further support; open discussions (25 minutes); closing - Follow-up meeting total time: 1 hour 10 minutes |
| **Workshop 5** | | | | |
|  | Simplify data collection | - To simplify data collection | - There are many ways of collecting and analyzing qualitative data - Issues related to reliability and validity of the data should be taken into account when using rapid and simple data collection methods - It is also important to reflect on the sample and potential sample size, as well as the influence of the team on the data in particular when more rapid data collection methods are used, which may not be as rigorous as traditional qualitative data collection - Reflections are best performed jointly with the social listening team - Checklists can be developed to discuss issues related to the reliability and validity of the data - It should be kept in mind that there are always compromises one must make when balancing between resources and scientific rigor - It is enough when teams strive to use the best possible methods available to them | - Welcome and agenda (5 minutes) - PowerPoint presentation: data recording (20 minutes) - Group work of frameworks that can facilitate data recording in your social listening process (20 minutes) - Reflections on the group work (20 minutes) - PowerPoint presentation: using frameworks (20 minutes) - Group work: types of frameworks that can facilitate analysis in a social listening project (20 minutes) - Reflections on the group work (20 minutes) - Open discussion, question and answer session, and closing (10 minutes) - Total time: 2 hours 15 minutes |
|  | Expected outcome | - Teams are able to simplify their data collection methods while ensuring rigor | - Teams are able to simplify their data collection methods while ensuring rigor | - Teams are able to simplify their data collection methods while ensuring rigor |
| **Workshop 6** | | | | |
|  | Simplify data analysis | - To simplify data analysis by and the recording the data | - The use of a conceptual framework makes data collection and entry simpler - There are many frameworks available - One can also design a framework that fits the purpose of the social listening exercise (misinformation and motivation) - Recording of data can be simplified; for example, by using digital platforms Microsoft Teams or chatbots or manual aids such as color-coding, mind maps, and sticky notes | - Welcome and agenda (10 minutes) - PowerPoint presentation: tips for simplifying analysis (20 minutes) - Group work: the type of framework social listening projects could benefit from and how one could start coding them (20 minutes) - Reflections on the group work (20 minutes) - PowerPoint presentation: tips for rapid recording of the data (10 minutes) - Open discussion and closing; closing (20 minutes) - Total time 1 hour 40 minutes |
|  | Expected outcome | - Teams are able to simplify their data analysis methods while ensuring rigor | - Teams are able to simplify their data analysis methods while ensuring rigor | - Teams are able to simplify their data analysis methods while ensuring rigor |
