# Supplementary material for: Social Listening to Enhance Access to Appropriate Pandemic Information Among Culturally Diverse Populations: Case Study From Finland
Source: JMIR Infodemiology. 2022 Jul 8;2(2):e38343. doi: 10.2196/38343 (PMC10014086; doi:10.2196/38343)
Supplement: Multimedia Appendix 2 [file infodemiology_v2i2e38343_app2.docx]

**Multimedia Appendix 2.** Resources for trainees.

| **Resources**   - WHO early IA supported response with social listening (https://whoinfodemic.citibeats.com/?cat=fYJ1oBNEUQtfbExrkGvsyr) - UNICEF Risk Communication and Community Engagement Strategy to Address the COVID-19 Infodemic (https://www.liebertpub.com/doi/full/10.1089/hs.2020.0226) - COVID-19 Facebook group poll results from (https://www.liebertpub.com/doi/10.1089/pop.2020.0189ed) - Data Collection and Analysis in Parallel to Share Emerging Findings in “Real-Time” from the U (https://journals.sagepub.com/doi/pdf/10.1177/1049732320951526) - The COVID-19 vaccines rush: participatory community engagement matters more than ever (https://www.thelancet.com/action/showPdf?pii=S0140-6736%2820%2932642-8) - U report (https://ureport.in/about/) - Community engagement for COVID-19 prevention and control: rapid evidence synthesis (https://gh.bmj.com/content/5/10/e003188) - COVID 19 risk perception monitoring system in Finland (https://www.who.int/docs/default-source/coronaviruse/risk-comms-updates/im-posters/poster-finland.pdf?sfvrsn=774eced4_5) - Understanding coronavirus disease (COVID-19) risk perceptions among the public to enhance risk communication efforts: a practical approach for outbreaks, Finland, February 2020 (https://www.eurosurveillance.org/content/10.2807/1560-7917.ES.2020.25.13.20003171) - Finnish Institute for Health and Welfare materials for pandemic-related risk communication (in Finish) - viestinnän materiaalipankki (https://thl.fi/fi/web/infektiotaudit-ja-rokotukset/ajankohtaista/ajankohtaista-koronaviruksesta-covid-19/materiaalipankki-koronaviruksesta) - koronatietoa eri kielillä (https://thl.fi/fi/web/infektiotaudit-ja-rokotukset/ajankohtaista/ajankohtaista-koronaviruksesta-covid-19/materiaalipankki-koronaviruksesta/koronatietoa-eri-kielilla) - sosiaalisen median toimintapolitiikka (https://thl.fi/fi/ajankohtaista/sosiaalinen-media/sosiaalisen-median-toimintapolitiikka) - WHO 2022 (https://www.who.int/ihr/publications/risk_communications/en/) - VNK:n projekti: Käyttäytymistieteellinen näkökulma vahvemmin esiin koronakriisin hoidossa (https://valtioneuvosto.fi/-/10616/kayttaytymistieteellinen-nakokulma-vahvemmin-esiin-koronakriisin-hoidossa-) - The behavior change wheel: A new method for characterizing and designing behavior change interventions (https://implementationscience.biomedcentral.com/articles/10.1186/1748-5908-6-42) - Factcheck.org. Debunking false stories (https://www.factcheck.org/fake-news/) - Controlling the spread of misinformation Psychologists’ research on misinformation may help in the fight to debunk myths surrounding COVID (https://www.apa.org/monitor/2021/03/controlling-misinformation) - The COVID-19 Vaccine Communication Handbook (https://osf.io/f6a48/) - Vaccine misinformation management field guide (https://vaccinemisinformation.guide/) - Three Cs model for vaccine hesitancy (https://www.researchgate.net/figure/Three-Cs-model-of-vaccine-hesitancy_fig1_275278401) - WHO EURO COVID-19 fatigue framework (https://apps.who.int/iris/bitstream/handle/10665/335820/WHO-EURO-2020-1160-40906-55390-eng.pdf) - COM-B to understand vaccine hesitancy (https://www.local.gov.uk/our-support/coronavirus-information-councils/covid-19-service-information/covid-19-vaccinations/behavioural-insights/resources/com-b-template) - Thematic analysis. Striving to meet the trustworthiness criteria (https://journals.sagepub.com/doi/full/10.1177/1609406917733847) - Using thematic analysis in psychology (https://www.tandfonline.com/doi/abs/10.1191/1478088706qp063oa) |
| --- |
